# Exploring the Impact of Personality Traits on Response to Anti-CGRP Therapies: Insights from a Pilot Study

**DOI:** 10.3390/neurosci7040077

**Published:** 2026-07-06

**Authors:** Chiara Zilli, Giada Giuliani, Giulio Tancredi, Mariangela Fratino, Benedetta Pitzalis, Marta Altieri

**Affiliations:** 1 Department of Human Neurosciences, Sapienza University of Rome, 00185 Rome, Italy; chiara.zilli@uniroma1.it (C.Z.); giada.giuliani@uniroma1.it (G.G.); mariangela.fratino@uniroma1.it (M.F.); benedetta.pitzalis@uniroma1.it (B.P.); 2 Department of Pediatrics, University of Rome “Tor Vergata”, 00133 Rome, Italy; giulio.tancredi@ptvonline.it

**Keywords:** chronic migraine, CGRP monoclonal antibodies, personality traits, treatment response

## Abstract

Background: Monoclonal antibodies targeting the calcitonin gene-related peptide (CGRP) pathway represent an effective preventive treatment for chronic migraine, including in patients with multiple prior therapeutic failures. However, a substantial proportion of patients exhibit a suboptimal or delayed response. The role of personality traits in modulating treatment response remains poorly understood. Methods: In this prospective observational study, adults with chronic migraine received anti-CGRP monoclonal antibodies and were followed for 12 months. Monthly headache days (MHDs), monthly acute medication use (MAM), and disability scores (MIDAS and HIT-6) were recorded at baseline and at 3, 6, and 12 months. Responders were defined as patients achieving a ≥50% reduction in monthly headache days (MHDs) compared with baseline. Changes in disability measures were evaluated as secondary clinical outcomes. Personality traits were assessed using the Millon Clinical Multiaxial Inventory-III. Results: Thirty-eight patients were included, of whom 71% had medication overuse headaches. Responder rates increased over time (31.6% at 3 months and 39.5% at 12 months). Histrionic traits (37.5% vs. 6.7%, *p* = 0.048) and dysthymic features (21.4% vs. 0%, *p* = 0.046) were significantly more prevalent among non-responders. Conclusions: Histrionic and dysthymic personality features were associated with a lower likelihood of response to anti-CGRP therapy in patients with chronic migraine. Given the exploratory design, limited sample size, and absence of correction for multiple comparisons, these findings should be considered preliminary and hypothesis-generating. Further studies in larger cohorts are needed to confirm these observations.

## 1. Introduction

Migraine is a neurological disorder characterized by recurrent attacks of moderate to severe headache, frequently associated with a range of accompanying symptoms. These may include sensory hypersensitivities such as photophobia, phonophobia, osmophobia, and allodynia; gastrointestinal symptoms such as nausea and vomiting; and broader systemic features including fatigue, cognitive slowing, and impaired concentration. It is the most common primary headache disorder and a leading cause of years lived with disability worldwide, particularly among individuals under 50 years of age [1].

The advent of monoclonal antibodies (mAbs) targeting the calcitonin gene-related peptide (CGRP) pathway has represented a major advancement in preventive treatment, demonstrating efficacy and safety even in patients with chronic migraine (CM) and multiple prior therapeutic failures. Nevertheless, up to one-third of patients experience an inadequate or delayed response [2].

Several clinical factors—including medication overuse headache (MOH), daily headache, obesity, and central sensitization—have been associated with poorer outcomes. In addition, psychiatric disorders can amplify pain perception, disrupt sleep, and enhance central sensitization, thereby reducing the effectiveness of preventive therapies for migraine. Patients with these comorbidities may therefore experience a reduced or slower response to anti-CGRP mAbs [2,3].

Among psychiatric comorbidities, mood and anxiety disorders are the most prevalent in individuals with migraine. Major depressive disorder shows a strong and consistent association, especially in patients with CM and migraine with aura, with epidemiological evidence indicating a significantly elevated risk compared to the general population. In addition, bipolar spectrum disorders appear to occur more frequently among migraine sufferers, suggesting a possible shared pathophysiological or genetic vulnerability. Anxiety disorders are also highly represented, with prevalence rates several times higher than those observed in non-migraine populations; notably, generalized anxiety disorder and panic disorder are among the most reported conditions [4].

These comorbidities negatively affect migraine-related disability, worsening quality of life and daily functioning. They also increase the burden between attacks due to anticipatory anxiety and avoidance behaviors. Furthermore, mood disorders—particularly major depression—are important risk factors for migraine chronicization, with severity directly linked to higher risk.

Beyond psychiatric diagnoses, even without meeting the criteria for personality disorders, patients with migraine frequently exhibit maladaptive personality traits, such as emotional dysregulation, maladaptive response to stress and ineffective coping strategies [5].

The notion that migraine might be associated with distinctive personality traits arose from repeated clinical observations of common behavioral tendencies among affected individuals. Migraine patients exhibited a propensity for high personal standards and great meticulousness, often accompanied by a preference for structure and control, as well as a marked goal orientation. These observations contributed to the first attempts to delineate a characteristic psychological profile in this population [6].

Previous evidence suggests that non-responders to anti-CGRP mAbs may show a higher tendency toward depressive symptoms and anhedonia. However, the specific contribution of personality traits to treatment response remains insufficiently explored [7].

The present study aims to address this gap by evaluating the potential impact of personality traits on treatment response in a real-world cohort of patients with treatment-resistant CM.

## 2. Methods

### 2.1. Study Design and Participants

Patients with a diagnosis of CM according to the International Classification of Headache Disorders 3rd edition were consecutively recruited at the Headache Center of Sapienza University between January 2024 and March 2025. All patients received anti-CGRP mAbs for 12 months. Eligibility criteria included adults with CM diagnosis; exclusion criteria comprised psychiatric disorders.

Treatment selection (erenumab, fremanezumab, galcanezumab) was based on clinical judgment and availability.

### 2.2. Clinical Assessment

Evaluations were performed at baseline and at 3, 6, and 12 months. Monthly headache days (MHDs) and monthly acute medications (MAMs) were recorded using headache diaries. Disability was assessed with the migraine disability assessment test (MIDAS) and headache impact test-6 (HIT-6) scores. Responders (Rs) were defined as patients achieving a ≥50% reduction in monthly headache days compared with baseline. MAMs, MIDAS, and HIT-6 were evaluated as secondary outcome measures.

### 2.3. Personality Assessment

Personality traits were evaluated at baseline using the Millon Clinical Multiaxial Inventory-III (MCMI-III), which provides a dimensional evaluation and differentiates between clinically significant personality traits (scores ≥ 75) and full personality disorders (scores ≥ 85) [8].

The MCMI-III was administered, based on two assumptions: an individual’s personality traits are stable over time and can therefore be reliably studied with a single administration of the test; the anti-CGRP mAbs, being specific for migraine, do not influence the subject’s personality traits or emotional profile. Individuals meeting criteria for full personality disorders were excluded from the study. This approach supports our focus on subthreshold personality characteristics rather than categorical psychiatric diagnoses.

The relationship between personality traits and treatment response was analyzed between Rs and non-responders (NRs).

### 2.4. Statistical Analysis

Continuous variables were presented as mean ± standard deviation (SD) or median with interquartile range (IQR), as appropriate. Group comparisons were performed using Student’s *t*-test or Pearson’s chi-square test. Associations between variables were explored using Kendall’s tau-β correlation. A *p*-value of <0.05 was considered statistically significant. Longitudinal changes in MHDs were additionally assessed using repeated-measures analysis, with time as the within-subject factor and responder status as the between-subject factor. Given the limited sample size, no correction for multiple comparisons was applied.

## 3. Results

The study population included 30 females and 8 males, with a mean age of 48 ± 12 years. No significant differences in age, sex, or treatment type (erenumab, fremanezumab, galcanezumab) were observed between Rs and NRs. Mean disease duration was 33.8 ± 13 years, and 27 patients (71%) had MOH. Patients were treated with erenumab (21%), fremanezumab (34%), or galcanezumab (45%).

Baseline MHDs were 20.3 ± 8, MAMs 18.9 ± 13, and MIDAS 79.1 ± 55.3.

At t-3, MHDs were 10.9 ± 8.8, MAMs were 6.5 ± 7.6, and MIDAS score was 28.3 ± 29.8.

At t-6, MHDs were 9 ± 7.6 and MAMs 5.9 ± 6.7, and MIDAS score was 31.2 ± 30.7.

At t-12, MHDs were 9.2 ± 7.7 and MAMs 5.9 ± 6.7, and MIDAS score was 31.4 ± 31.7 (Table 1).

Responder rates progressively increased: 31.6% at 3 months, 36.8% at 6 months, and 39.5% at 12 months, suggesting the presence of late responders. We observed changes in the parameters studied compared to baseline even in the NR group. HIT-6 scores showed greater reduction among responders compared to non-responders (Table 2).

**Figure 1:**
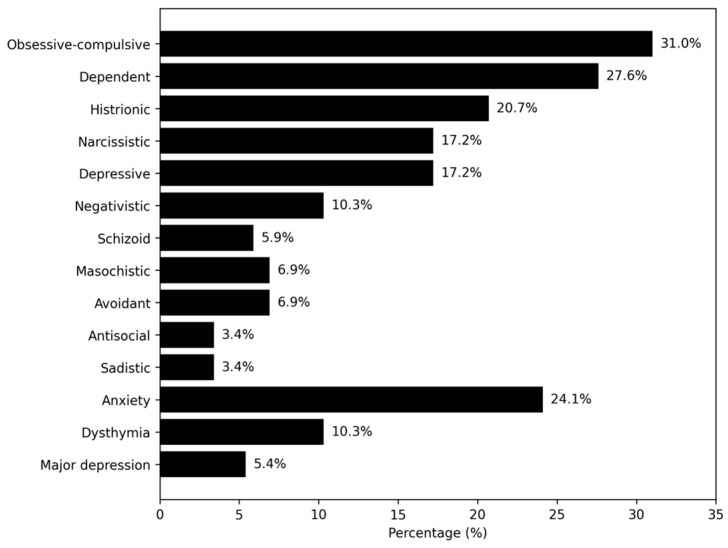
Distribution of personality traits within the cohort.

The most prevalent personality traits were obsessive-compulsive (31%), dependent (28%), and histrionic (21%) (Figure 1).

**Figure 2:**
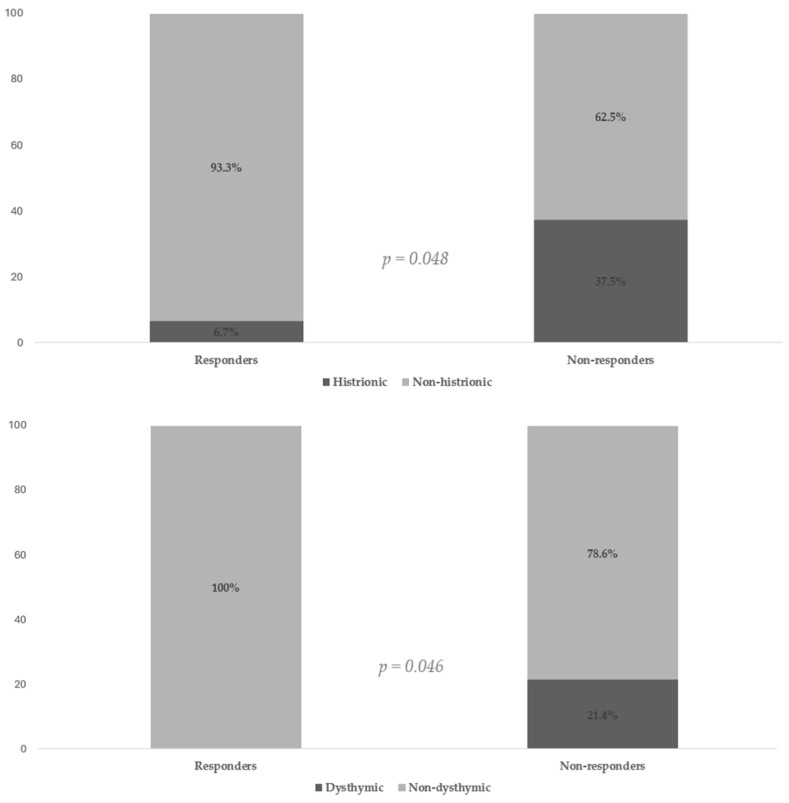
Comparison of histrionic and dysthymic traits between responders and non-responders.

When stratified by treatment response, non-responders showed a higher prevalence of histrionic traits (37.5% vs. 6.7%, *p* = 0.048) and dysthymic features (21.4% vs. 0% *p* = 0.046), suggesting a potential association between these traits and reduced responsiveness to anti-CGRP therapy (Figure 2).

Longitudinal repeated-measures analysis (Friedman non-parametric test for paired observations across four time points) showed a significant effect of time for all clinical outcomes. Monthly headache days (MHDs) significantly decreased from 19.8 at baseline to 9.3 at 12 months (*p* < 0.0001). Similarly, monthly acute medication use (MAM) decreased from 21.9 to 6.7 days/month (*p* < 0.0001). Disability measures also showed significant improvement over time, with MIDAS scores decreasing from 72.2 to 29.6 (*p* < 0.0001) and HIT-6 scores from 64.9 to 56.4 (*p* < 0.0001).

## 4. Discussion

The present study provides real-world evidence supporting the sustained effectiveness of anti-CGRP mAbs in patients with treatment-resistant CM. Nearly 40% of patients achieved a clinically meaningful reduction in headache frequency at 12 months. Notably, the progressive increase in responder rates over time suggests the presence of delayed responders and supports the continuation of therapy beyond the early treatment phase, particularly in complex clinical populations. Recent studies have emphasized the importance of new treatments and the possibility of significant improvement also after a partial response in the first trimester [9].

The influence of specific comorbidities also should not be overlooked, such as the impact of psychological aspects on treatment outcomes.

Psychological factors may play an important role in modulating treatment outcomes. Psychiatric disorders are known to promote migraine chronification, and dysregulation of serotoninergic, dopaminergic, and noradrenergic systems—commonly observed in patients with depression and anxiety—may influence the response to anti-CGRP therapies [10,11].

According to a recent study, non-responders to anti-CGRP mAbs seemed to have a higher tendency toward depressed mood, based on assessment with Personality Inventory for DSM V [7]. The role of personality traits on migraine preventives is more debated. Avoidant, dependent, or obsessive-compulsive personality patterns have been linked to a lesser response to anti-CGRP mAbs therapies [12].

At the same time, in a previous study investigating the personality traits of migraine patients using the Minnesota Multiphasic Personality Inventory (MMPI), the authors suggested that headache cases can develop different personality characteristics as coping mechanisms against recurrent pain. The MMPI scales of hypochondriasis, depression, and hysteria—defined as the “neurotic triad”—were associated with traits such as excessive concern about health, sleep and attention problems, low self-esteem, and pessimism. Individuals with high scores often reported strong physical symptoms under stress. In terms of personality traits between episodic migraine (EM) and CM case groups, the histrionic personality trait was significantly higher. CM patients tended to show higher neuroticism and social introversion than EM patients [4].

A key finding of this study is the observed association between specific personality traits—namely histrionic and dysthymic features—and a reduced likelihood of treatment response. These results suggest that psychological and personality-related factors may contribute to variability in therapeutic outcomes, beyond traditional clinical predictors. In these patients, impaired coping strategies could promote the progression toward more disabling forms of the migraine, characterized by higher frequency and intensity of attacks. The long history of migraine, often marked by multiple therapeutic failures, could exacerbate feelings of frustration and discomfort while the improper use of analgesic drugs could facilitate the development of MOH.

From a pathophysiological perspective, this association may reflect the complex interplay between neurobiological and psychosocial mechanisms underlying migraine. Personality traits do not always directly correlate with depression severity, suggesting migraine itself may contribute to psychiatric conditions. CM patients generally have higher levels of anxiety, depression, and certain personality traits (e.g., histrionic features) than EM patients. Migraine is also linked to brain changes (e.g., in the prefrontal cortex), increased anger tendencies, and traits like perfectionism and neuroticism. Overall, migraine—especially chronic forms—is strongly associated with distinct personality patterns and psychiatric features, which may worsen with disease severity and medication overuse [8].

Furthermore, individuals with maladaptive personality traits may exhibit heightened emotional reactivity, altered stress responses, and dysfunctional coping strategies, all of which can amplify pain perception and contribute to central sensitization.

In patients with specific personality traits, neurotransmitters other than CGRP and the mechanism of central sensitization could play a role. CGRP is finely involved in both pain and mood regulation; however, higher levels of this neuropeptide do not necessarily result in greater response to anti-CGRP medications, as observed in patients with depressive disorders [7].

Importantly, migraine is increasingly recognized as a multifactorial disorder involving multiple neurochemical pathways beyond CGRP signaling. In patients with prominent psychological or personality-related features, non-CGRP mechanisms—such as glutamatergic transmission or stress-related neuroendocrine pathways—may play a more dominant role, potentially limiting the clinical effectiveness of CGRP blockade alone. This hypothesis may partially explain the reduced responsiveness observed in patients with histrionic or dysthymic traits in our cohort. The increasing understanding of migraine pathophysiology and research on drugs targeting different molecules (e.g., glutamate, substance P) will help shed light on these issues.

Nevertheless, the observed associations should be interpreted cautiously. The statistical significance of some findings was borderline, and no correction for multiple comparisons was applied. Therefore, the results should be regarded as exploratory and hypothesis-generating rather than confirmatory.

Personality traits refer to enduring patterns of thoughts, emotions, and behaviors that characterize individuals. These traits are defined by three core features: consistency across situations, stability over time, and the presence of individual differences. They influence how individuals respond to environmental stimuli and represent relatively stable personal characteristics [13]. Importantly, such traits can be reliably assessed through a single administration of standardized self-report measures [14]. Several studies have investigated the association between personality traits and migraine. Notably, some studies have reported a higher prevalence of histrionic traits in patients with chronic migraine compared to healthy controls [6].

From a clinical standpoint, these results underscore the importance of a comprehensive, multidimensional assessment of patients with CM. The integration of psychological evaluation into routine clinical practice may help identify individuals at risk of suboptimal response and guide more tailored therapeutic strategies.

It is important to note that, in clinical practice, a variety of tools are used for psychological and personality assessment, resulting in considerable heterogeneity across findings. The lack of standardized assessment method remains a relevant limitation, despite the well-established presence of psychiatric comorbidities in patients with migraine.

Following a comprehensive evaluation, a multidisciplinary approach may be essential to optimize treatment response. Psychological interventions are increasingly recognized as effective components of migraine management, contributing to improved quality of life. These strategies may include the integration of pharmacological treatments with targeted psychological interventions, such as cognitive behavioral therapy, mindfulness-based approaches, or short-term psychodynamic psychotherapy, all of which have demonstrated efficacy in enhancing coping strategies and reducing migraine-related disability [15,16].

Furthermore, especially in patients with psychiatric disorders, it is crucial to provide clear and accurate information regarding realistic treatment expectations and potential adverse events, which may be perceived in an amplified way, leading to premature discontinuation of effective therapy. In this context, individualized pharmacological strategies—such as dose optimization, switching between different anti-CGRP agents, or combining therapies (e.g., with onabotulinumtoxinA or mood-stabilizing agents)—may be especially relevant in patients with complex clinical and psychological profiles.

Despite these considerations, caution is warranted in interpreting the present findings. The observational design limits causal inferences, and factors such as the high prevalence of MOH, treatment heterogeneity, and long disease duration may have influenced the results. Future studies incorporating biological, psychological, and behavioral variables within larger, multicenter cohorts are needed to better elucidate the mechanisms underlying treatment response variability.

## 5. Limitations

The small sample size, single-center design, and lack of correction for multiple comparisons limit the generalizability of the findings. In addition, relevant biological and psychosocial variables were not systematically assessed. The absence of multivariate analysis further limits the ability to control for potential confounders, underscoring the need for larger, multicenter studies to confirm these preliminary observations.

Moreover, the limited characterization of personality traits and the absence of structured psychiatric interviews represent additional methodological limitations. Furthermore, potential confounding factors, including medication overuse headache, disease duration, and treatment heterogeneity, were not formally controlled for through multivariable analyses.

## 6. Conclusions

In conclusion, this pilot study suggests that histrionic and dysthymic personality features may be associated with a lower likelihood of response to anti-CGRP therapies in patients with chronic migraine. Given the exploratory design, limited sample size, absence of correction for multiple testing, and potential residual confounding, these findings should be considered preliminary and hypothesis-generating. Nevertheless, they provide initial evidence supporting the potential role of personality traits as modifiers of treatment response and justify further investigation in larger prospective studies.

## Figures and Tables

**Table 1:** The change in MHDs, MAMs, MIDAS and HIT-6 over time in our population.

	t-0	t-3	t-6	t-12
MHDs	20.3 (8)	10.9 (8.8)	9 (7.6)	9.2 (7.7)
MAMs	18.9 (13)	6.5 (7.6)	5.9 (6.7)	5.9 (6.7)
MIDAS	79.1 (55.3)	28.3 (29.8)	31.2 (30.7)	31.4 (31.7)
HIT-6	66.1 (6.5)	57.9 (9.2)	55.8 (10.8)	58.8 (9.8)

**Table 2:** The different change over time in the MHDs, MAMs, MIDAS and HIT-6 scores, both in R and NR group.

	t-0	t-3 Rs	t-6 Rs	t-12 Rs	t-3 NRs	t-6 NRs	t-12 NRs
MHDs	20.3 (8)	6 (4.9)	5.9 (3.8)	4.8 (3.5)	17.6 (9)	16.7 (9.2)	14.2 (8.1)
MAMs	18.9 (13)	5 (5.4)	4.6 (2.9)	4.1 (3.3)	8.4 (9.8)	9.2 (11.3)	8.1 (8.8)
MIDAS	79.1 (55.3)	18 (20)	25.1 (31.9)	30.3 (35.6)	42.6 (35.4)	46 (22.3)	32.6 (27.6)
HIT-6	66.1 (6.5)	55.7 (9.9)	53.1 (11.3)	56.6 (10.8)	60.9 (7.2)	62.5 (5.6)	61.3 (10.8)

## Data Availability

The original contributions presented in this study are included in the article. Further inquiries can be directed to the corresponding author(s).

## Abbreviations

CGRP	calcitonin gene-related peptide
CM	chronic migraine
EM	episodic migraine
HIT6	headache impact test-6
mAbs	monoclonal antibodies
MAMs	monthly acute medications
MCMI-III	Millon Clinical Multiaxial Inventory-III
MHDs	monthly headache days
MIDAS	migraine disability assessment test
MOH	medication overuse headache
NRs	non-responders
Rs	responders
